# DO RELIGION, CULTURE, RACE, OR ETHNICITY PLAY A ROLE IN CHOOSING PEG TUBE AT THE END OF LIFE?

**DOI:** 10.1093/geroni/igad104.3288

**Published:** 2023-12-21

**Authors:** Isabella Park, Guisela Hernandez, Frederick Lambert, Elisabeth Loccisano, Christian Nouryan, Catherine Cho, Asha Gajraj, Edith Burns

**Affiliations:** Northwell Long Island Jewish Forest Hills, Forest Hills, New York, United States; Northwell Health, Valley Stream, New York, United States; Northwell Health, Forest Hills, New York, United States; Northwell Health, Valley Stream, New York, United States; Northwell Health, Manhasset, New York, United States; Northwell Health, Manhasset, New York, United States; Northwell Health, Manhasset, New York, United States; Zucker School of Medicine at Hofstra/Northwell, Manhasset, New York, United States

## Abstract

Although there is substantial evidence that Percutaneous Endoscopic Gastrostomy (PEG) causes significant morbidity and does not change mortality for patients with advanced dementia it continues to be used. We describe a quality initiative of Palliative Care Consultation (PCC) in conjunction with any new PEG evaluation. EMR review and interview with patient (and/or proxy) being evaluated for first-time PEG in two community hospitals within large health system in demographically diverse major metropolitan area. PCC teams provided consistent, culturally/religiously, language-sensitive education regarding PEG, conducted interviews about subsequent decision. Post-initiative PEG placement dropped from >75% to 17.6%, 76 patients received PCC with PEG evaluation. Average age 83.5 (range: 56-108), 55% female. Religion: 54% Christian, 7% Muslim, 10% Jewish, 11% non-religious, 18% other; 46% White, 37% Black, 8% Asian, 8% Hispanic. Common evaluation reasons: difficulty swallowing (30%), low PO intake (30%). Majority of patients had dementia (71%), stroke (18%), Parkinson’s (8%). Most decision-makers (66%) hadn’t received prior counseling regarding alternate nutrition options. After PCC, 17.6% chose PEG, 79.7% careful hand feeding, 2.7% remained unsure. Those choosing PEG mostly desired “guaranteed” nutrition despite risks (10/13), and/or a trial seeking patient recovery (5/13). There were no apparent differences in race/ethnicity, age, sex, or religion that distinguished those electing PEG. PCC initiative to support patients and families “choosing wisely” around PEG placement for those with advanced illness has resulted in significant reduction. Decision-makers mostly cited wish to guarantee nutrition while some hoped for patient recovery. Religious affiliation and sex were not cited as reasons in this group.

